# Impacts of Cultural Diversity on Carbon Emission Effects: From the Perspective of Environmental Regulations

**DOI:** 10.3390/ijerph17176109

**Published:** 2020-08-22

**Authors:** Ying-jie Song, Fu-wei Ma, Jing-ya Qu

**Affiliations:** 1 School of Finance, Shandong Technology and Business University, Yantai 264005, Shandong, China; songyingjie@sdtbu.edu.cn (Y.-j.S.); 2019410030@sdtbu.edu.cn (F.-w.M.); 2 Cooperative Innovation Center for Finance Serves for Transformation and Upgrading, Shandong Technology and Business University, Yantai 264005, Shandong, China

**Keywords:** cultural diversity, environmental regulation, environmental preference, carbon emission effect

## Abstract

Cultural diversity is an issue not considered too often in traditional research on the influencing factors of carbon emission reduction to give full play to the effective participation of micro subjects in environmental regulation and to achieve the carbon emission reduction target. Aiming at the cultural diversity of micro subjects, this paper introduces the provincial dynamic index of cultural diversity and, from the perspective of environmental regulation, combines environmental regulation types such as energy regulation, economic regulation, and administrative regulation, to empirically study the impact of cultural difference on carbon emission reduction. We found that cultural diversity had a significant negative impact on carbon emission effects, and there is a one-way Granger causality between the two. Cultural diversity and environmental regulations exerted a synergistic impact on carbon emission effects. Through specific mechanism tests, the intermediary effect of environmental regulations was confirmed. Cultural diversity influenced carbon emission effects through the mediation of environmental regulations. From the perspective of the refined characteristics of different regions, possible cultural diversity in the southern region and regional energy consumption characteristics significantly affected carbon emission effects. On the basis of the conclusions reached in this empirical research, we put forward the following policy suggestions: emphasis should be placed on the function of culture and other non-institutional factors in the practice of environmental regulations; bottom-up environmental protection incentives must be strengthened, and required expression channels should be perfected; the role of various environmental regulations must be given full play in the process of carbon emission reduction.

## 1. Introduction

In the past 40 years, China’s economy has experienced rapid growth, and the energy industry, especially the energy-intensive industry, played a central role in the economic growth. With a drastic rise in energy consumption, China surpassed the United States as the world’s greatest carbon emitter in 2007 (Derived according to the IEA data). Effectively coping with global warming and curtailing carbon emission are imperative issues that China currently faces. To solve this issue, the Chinese government formulated a series of dual-control emission reduction targets to reduce carbon emission intensity and achieve peak carbon emissions. In 2015, China promised to the world that it will reach the CO_2_ emission peak by 2030 and achieve unit gross domestic product (GDP) CO_2_ emissions 60–65% lower than in 2005. To achieve these goals, the Chinese government introduced a series of environmental regulation policies on energy, including the formulation of a nationwide air pollution control plan, reduction of fossil fuel consumption, and development of new clean energy sources. However, under the background of proactive fiscal policy and prudent monetary policy, China’s economy will still maintain a medium–high growth thanks to the promotion of the “three carriages” [1,2]. Economic growth can inevitably bring about a rise in energy consumption in its wake. Coordinating this contradiction is an important issue that concerns not only China but also the whole world.

In the context of a series of environmental problems, such as global warming and environmental pollution, carbon emission reduction and environmental governance became the focus of global concern [3]. Given the public’s good attitude toward carbon emission and its regulations, governments can implement corresponding regulations through administrative and economic means, such as improving laws and regulations, raising emission standards, guiding energy consumption restructuring, and strengthening financial support to hold the scale of carbon emission at bay [4,5,6]. Regarding the influencing factors of carbon emission reduction, traditional literature mostly focuses on the traditional environmental regulation factors with the government as the main body. It is believed that there is a certain relationship among greenhouse gas emission (related to energy use), population, development level, energy use efficiency, per capita income, energy intensity, and carbon emission factors per unit energy consumption [7,8]. In addition, China’s more significant carbon emissions factors include economic growth, increased income, energy intensity, energy structure, industrial structure, and gross output value [9,10]. However, governments are not the only subject involved in environmental regulations; the effectiveness of such regulation hinges on the bottom-up participation of micro subjects, such as the public, enterprises, and other organizations [11]; that is, a large number of stakeholders participating in microeconomic activities and their highly differentiated decisions promote the effective operation of environmental regulations [12,13]. The above literature rarely considers the influence of micro subjects.

During this process, governments generally adopted the practice of formulating environmental regulation policies in a “top-down” manner for a long time [14,15], as citizens and other micro subjects are believed to lack motivation to engage in environmental regulations in line with the egoism mentality [16]. This kind of policy system is often decided by central governments and implemented by local governments [17], as it allows for quick decisions, effective fund pooling, and joint prevention and governance across regions nationwide. However, issues exist such as the neglection of the “bottom-up” feedback (citizens and other micro subjects) mechanism during the process of environmental regulations and the lack of differentiated considerations for local environmental governance. The formulation of macro policies essentially originates from the actual needs of citizens and other micro subjects. As micro individuals, citizens can display their environmental preferences through their energy consumption structure and environmental protection participation [18], ultimately impacting environmental regulation policies. A sound environmental governance framework and realization of the ultimate emission reduction target should be based on the joint participation of the government, society, and citizens [19].

The theory of public participation in ecological environment regulation can be traced back to that of “voting with feet” [20]. Residents transmit public preference information with their migration to promote their region and, thus, improve its public services [21,22]. With the proliferation of research, the relationship between citizens and other social micro subjects’ demand preferences and environmental regulations is receiving increasing concern. Social micro subjects are closely related to their surrounding ecological environment. A group or organization composed of heterogeneous micro subjects may also exhibit a collective heterogeneity in terms of the ecological environment. For example, citizens’ religious belief diversity may lead to different responses to ecological environment issues [23]. Public environmental preferences can influence carbon emission effects via “bottom-up” environmental policymaking [24]. Among them, cultural characteristics are important indexes that affect public environmental preferences [25,26]. Research shows that cultural diversity is a potential barrier to the low-carbon transformation and can mediate the ultimate effect of carbon emissions [27]. Citizens have different cultural backgrounds, and cultural attributes reflect the energy consumption characteristics and environmental preferences of micro subjects. Due to the vast territory and numerous nationalities in China, cultural diversity, defined as the unique individual attributes of citizens living in an area with obvious regional diversity, can reflect the unique energy consumption demand characteristics of the region, allowing the formation of similar environmental preferences. When formulating and implementing environmental regulations and energy policies, governments should fully consider citizens’ cultural characteristics [28,29,30,31]. At present, available studies on cultural diversity and carbon emission effects are limited, and a few recent studies only qualitatively analyzed the impact of cultural diversity on carbon emission effects [32,33], without verifying the impact of cultural diversity on carbon emissions at an empirical level, let alone exploring in depth the possible internal impact mechanism of environmental regulations, an intermediate variable, in the process of cultural diversity affecting carbon emissions.

The present study empirically examined the impact of culture, which is a non-institutional factor, on carbon emission effects and attempted to explore the logic and mechanism of a multi-party governance framework involving the government and the public. The contribution of this study lies in three aspects. Firstly, it empirically studied the effects of cultural diversity and environmental regulations on carbon emission effects. Secondly, it verified the preferences of micro subjects reflected in cultural diversity in terms of the mechanism of environmental regulations ultimately influencing carbon emissions. Thirdly, the dynamic index of cultural diversity based on dialect data was introduced to identify the impact of culture on emission reduction under the framework of China’s unified political system.

The structure of this paper is as follows: Section 1 introduces the relevant research background and sorts out the theoretical logic for the impacts of cultural factors and environmental regulations on carbon emissions in the current research. Section 2 explains the preparation for the empirical study and the construction of the empirical model and data sources. Section 3 empirically examines the various impacts of cultural diversity and environmental regulations on carbon emissions and further analyzes the joint influencing mechanism of cultural diversity and environmental regulations on carbon emissions. Section 4 provides the conclusion and policy analysis.

## 2. Empirical Preparation

### 2.1. Model Building

Based on the research content, this paper used the dynamic panel generalized method of moments (GMM) method to study the effects of cultural diversity on carbon emissions, which allows the heteroscedasticity and sequence correlation of random error terms; thus, the parameter estimator obtained is more effective than other parameter estimation methods. Although variables that may affect carbon emissions are controlled in the above approach, there may still be missing variables. In the field of environmental protection, the current carbon emissions are vulnerable to the impact of carbon emissions in the lag period. Compared with the traditional static panel model, the dynamic panel model controls the impact of the lag-phase carbon emissions on the current carbon emissions. The dynamic panel GMM can effectively avoid the deviation caused by endogenous problems. Dynamic GMM uses differential conversion data, which can effectively overcome the problem of missing or unobserved variables. It is generally believed that system GMM uses more information than differential GMM, and it can estimate individual effect coefficients. Therefore, system GMM can be considered to be more effective than differential GMM. Therefore, this article implements the more widely used system GMM estimation for analysis.

The basic panel data format of the model is as follows:

(1) $$I_{\mathrm{it}}=aP_{\mathrm{it}}^{b}A_{\mathrm{it}}^{c}T_{\mathrm{it}}^{d}e_{\mathrm{it}},$$

where *I* indicates the carbon emission effect, *P* indicates the size of the population, *A* indicates the degree of wealth, *T* indicates the level of technology, *a* is a constant term, *b* is the elasticity of population size, *c* is the elasticity of wealth degree, *d* is the elasticity of technology level, *t* indicates time, and *e* indicates the error term. Taking into account the time inertia of environmental regulation, on the basis of Equation (1), environmental regulation, cultural diversity, and carbon emission variables are added to adopt a dynamic panel model. The logarithmic form of the variables is as follows:

(2) $$\ln coe_{\mathrm{it}}=\alpha +\beta_{1}\ln coe_{i,t-1}+\beta_{2}\ln ger_{\mathrm{it}}+\beta_{3}\ln dc_{\mathrm{it}}+\beta_{4}\ln\chi_{\mathrm{it}}++\eta_{i}+\tau_{t}+\varepsilon_{\mathrm{it}},$$

where $\eta_{i}$ is the local fixed effect, $\tau_{t}$ is the time fixed effect, and $\varepsilon_{\mathrm{it}}$ is the residual term. In this model, *coe*_it_ represents carbon emissions, *ger*_it_ represents environmental regulations, *dc*_it_ represents provincial cultural diversity, and $\chi_{\mathrm{it}}$ represents other control variable sets that may affect carbon emissions.

### 2.2. Index Selection

#### 2.2.1. Core Variables

(1) Carbon emissions (*coe*_it_). With regard to the measurement of carbon emissions and with reference to the standard coal method of the Intergovernmental Panel on Climate Change (IPCC), the calculation formula of carbon dioxide emissions published by the IPCC was used to measure carbon dioxide emissions in various provinces in China. Eight energy sources, namely, coal, coke, crude oil, gasoline, kerosene, diesel oil, natural gas, and fuel oil, in different provinces were used as the end consumption. The consumption of these eight energy sources was multiplied by their respective carbon emission coefficients. Given the diversity in regional population, the per capita carbon emission in each province was used as the carbon emission measurement index.

(2) Environmental regulations (*ger*_it_). Multiple indicators exist to measure environmental regulations. With reference to classical practice in most studies, environmental regulations were divided into energy regulation (*ger*_it_-*N*), economic regulation (*ger*_it_-*E*), and administrative regulation (*ger*_it_-*A*) in this study [34,35]. However, there are many insufficient environmental regulation measures and policies for energy consumption, and it is difficult to find independent indicators to measure them. Energy regulation that is closely related to carbon emissions was selected as the main environmental regulation variable, represented by the energy intensity (energy consumption per unit GDP, a contrary indicator) of each province [36,37,38]. To further enhance the robustness of the results, the ratio of pollution discharge fees levied in a province in the current year to its GDP and the ratio of the number of environmental penalties imposed in a province in the current year to its GDP were used to represent economic and administrative regulations for comparative analysis, respectively.

(3) Cultural diversity (*dc*_it_). China exhibits significant regional differences in terms of culture. A few standards can be followed to measure cultural diversity among regions in China. With reference to the method of Xu et al. [39], the present study considered dialects as indicators of regional cultural diversity. As dialects contain information about the living habits of people in different regions, they can reflect the energy consumption needs and environmental preferences of micro subjects. Taking province as the analysis unit, we counted the number of dialects other than Mandarin Chinese in different provinces as a proxy variable to measure cultural diversity in different provinces and expressed it as *N*. To reflect the dynamic changes of cultural diversity among regions in terms of time, the annual population change coefficient of a province was introduced and dynamically optimized to measure the annual dynamic variation of cultural diversity among regions.

(3) dc=(1+λ)∗N,

where *N* indicates the number of dialects other than Mandarin Chinese in different provinces, and λ is the coefficient of population change in each province.

(4) $$\lambda =\frac{\mathrm{Permanent}\;\mathrm{residents}\;\mathrm{at}\;\mathrm{the}\;\mathrm{end}\;\mathrm{of}\;\mathrm{the}\;\mathrm{current}\;\mathrm{year}-\mathrm{Permanent}\;\mathrm{residents}\;\mathrm{at}\;\mathrm{the}\;\mathrm{end}\;\mathrm{of}\;\mathrm{previous}\;\mathrm{year}}{\mathrm{Permanent}\;\mathrm{residents}\;\mathrm{at}\;\mathrm{the}\;\mathrm{end}\;\mathrm{of}\;\mathrm{previous}\;\mathrm{year}}.$$

#### 2.2.2. Control Variables

With reference to relevant literature [40,41], this paper added other factors that affect the carbon emission effect to the regression equation.

(1) Economic development level (*gdp*_it_). The real GDP per capita of different regions (using the Consumer Price Index (CPI) to deflate the nominal variables with 2006 as the base period) was selected to measure the economic development level of different regions.

(2) Population density (*dp*_it_). The number of permanent residents in the unit jurisdiction area was used to indicate the population density.

(3) Industrial structure (*ins*_it_). The proportion of the added value of the secondary industry to GDP was used to measure the industrial structure.

(4) Energy consumption structure (*ecs*_it_). The proportion of coal consumption to the total energy consumption was used to indicate the energy consumption structure.

### 2.3. Data Sources

To rule out heteroscedasticity and ensure data stability, we adopted the natural logarithm of the data. To mitigate the influence of outliers on the regression results, we conducted the 1% and 99% Winsorization of all continuous variables and the positive management of contrary indicators. We used panel data of 30 provincial regions in mainland China from 2006 to 2015 for the follow-up empirical test. The number of Chinese sub-dialects came from the Large Dictionary of Chinese dialects, while GDP, added value of secondary industry, number of permanent residents, foreign direct investment, and other data were obtained from the China Statistical Yearbook (2007–2016) over the years. Moreover, CPI was used to deflate the nominal variables with 2006 as the base period. Specific variable descriptions are shown in Table 1.

## 3. Empirical Analysis

The empirical research was conducted in four stages. In the first stage, we performed the basic model regression. We initially analyzed the impacts of regional cultural diversity on carbon emissions. We then subdivided environmental regulations into energy, economic, and administrative regulations for comparative analysis. Moreover, the causal relationship between cultural diversity and carbon emissions was examined. The empirical results of the basic model and the results of causal analysis are shown in Section 3.1, respectively. In the second stage, we included the interaction term between cultural diversity and environmental regulations to investigate their combined effect on carbon emission effects. The empirical results and the interaction term model are presented in Section 3.2. In the third stage, due to the huge cultural diversity between south and north China and their energy consumption differences due to “winter heating” in the north, we further conducted an empirical test of the detailed regional characteristics of the south and the north. The empirical results are shown in Section 3.3. In the fourth stage, we analyzed the realization mechanism for the combined impact of cultural diversity and environmental regulations on carbon emissions. The empirical results of the mechanism test are provided in Section 3.4.

### 3.1. Basic Regression

We adopted the systematic GMM method in this study to avoid the issues of weak instrumental variables and potential endogeneity. According to the AR (1) and AR (2) tests in Table 2, each model had a first-order sequence autocorrelation, but no second-order sequence autocorrelation. The Sargan test showed that the instrumental variables selected by each model were exogenous and valid, and no model over-recognition was observed. The above tests revealed that the GMM estimation results were reliable and valid.

*ger*_it_-*N* in Table 2 shows the impacts of cultural diversity and environmental energy regulations on carbon dioxide emissions in various provinces in China. To further enhance the stability of our results, we used economic and administrative regulations to replace energy regulation indicators for a comparative analysis of robustness, as illustrated in the results of *ger*_it_-*E* and *ger*_it_-*A*, respectively. The estimation results revealed that energy, economic, and administrative regulations had significant inhibitory effects on carbon emissions. Per capita carbon dioxide emissions were reduced by 0.304 per unit increase in energy regulation intensity, indicating that carbon emissions can be effectively reduced by means of energy regulation policies focusing on advocating clean energy consumption, reducing the proportion of traditional fossil fuel consumption, and improving energy utilization rate. By comparing the regression coefficient, we can find that the influence of energy regulation variables on carbon emissions is stronger than economic regulation and administrative regulation. However, due to the limitations of variable design and research methods in the study, energy intensity as the proxy variable of energy regulation can only indirectly reflect the impact of government energy regulation policies from the side, and it is impossible to measure the specific role of energy regulatory policies in limiting carbon emissions. How to scientifically and accurately measure energy regulations and evaluate their impact on carbon emissions is the direction for our follow-up research. In terms of the empirical results of the basic model, cultural diversity did not exhibit a definite effect on carbon emissions. The coefficient in the economic regulation model was significant, but those in the energy and administrative regulation models were not. The coefficient signs in the three models were inconsistent and, thus, must be further studied empirically. As explained variables, the first-phase lagged variables of carbon emissions were all significant at the 1% level, indicating that carbon dioxide emissions have strong “inertia” characteristics; that is, the carbon emission level in the previous year can have a significant impact on the local carbon emission in the coming year. From the perspective of control variables, economic development level and population density had significant impacts on carbon emissions.

Table 3 shows that cultural diversity is not the Granger cause of carbon emissions, and the null hypothesis was rejected at the significance level of 5%. Carbon emissions are not the Granger cause of cultural diversity, and the null hypothesis was accepted. This indicates that there is a one-way causal relationship between cultural diversity and carbon emissions, and cultural diversity is the Granger cause of carbon emissions.

### 3.2. Combined Impact of Cultural Diversity and Environmental Regulations

In the above theoretical analysis, we discussed that environmental regulation behaviors may be influenced by residents’ environmental preferences, and both factors may generate a combined impact on carbon emissions. To verify the possible combined impact, we introduced interaction terms—cultural diversity and environmental regulations—to the empirical model and used the systematic GMM method for estimation. The estimation results are presented in Table 4.

Table 4 shows that the interaction terms among the three types of environmental regulation behaviors and cultural diversity were significantly positive, verifying that environmental regulations and cultural diversity jointly exert an adverse effect on carbon emissions. In the results of *ger*_it_-*N*, *ger*_it_-*E*, and *ger*_it_-*A*, cultural diversity exhibited an adverse effect on carbon the effective control of carbon emissions, which can be interpreted as that differences in citizens’ energy demand preferences reflected by cultural diversity contribute to different attitudes toward energy conservation and emission reduction in different regions, and a decrease in the identification of social public with the effect of social emission reduction weakened the effect of different types of environmental regulations on carbon emissions. Moreover, certain groups do not actively participate in the campaign for energy conservation and emission reduction, thereby weakening the realization of carbon emission targets through the free-riding effect [42,43]. Of course, it should be admitted that there are also many possible reasons. Residents in culturally diverse areas are living in a complex environment, and their demands for living conditions are more diversified. Therefore, they are faced with greater challenges in lobbying authorities to meet their own needs and better regulate the pollution industry in their areas. The underlying reasons that may cause the negative impact of cultural differences on carbon emissions need to be further studied. Note that the three types of environmental regulation behaviors showed significant effects on carbon emissions, but in different directions. Energy and economic regulations promoted carbon emission reduction, whereas administrative regulation undermined the effect of carbon emission reduction. One possible reason is that, in nature, energy regulation still mainly features economic regulation. Its regulatory role in carbon emissions may be easily realized through economic means, whereas administrative regulation is coordinated by administrative compulsion, which may yield undesired effects during the implementation process.

### 3.3. Empirical Analysis of South and North China

Due to the vast territory of China, a large cultural disparity exists between its south and north regions. Differences in energy consumption and carbon emissions are also observed, owing to the different “winter heating” patterns in both regions. We further took Qinling Mountains–Huaihe River as the boundary to conduct an empirical test of the south and the north in energy consumption. The results are shown in Table 5.

The empirical results indicated that, in the north, most environmental regulations, cultural diversity, and their interaction terms had no significant impact on carbon emissions, whereas, in the south, the impacts were consistent with the overall results of the whole country. Therefore, a huge difference exists between north and south China in terms of sensitivity to the impacts of cultural diversity and environmental regulations. The reasons may be that many ethnic groups can be found in the south, and the topography is mainly mountainous and hilly, with a huge fluctuation in terrain, as reflected in the great variety and difference of dialects and frequent occurrence of “a different dialect found within a ten-li distance”. Although many dialect areas exist in the north, basic communication can be realized in general. Based on the cultural diversity in terms of dialectal dimension between south and north China, the south has great degrees of discrimination and cultural diversity, and residents demand the government to extensively control carbon emissions. Therefore, compared with the northern region, cultural diversity and environmental regulations in the south have significant impacts on carbon emission reduction.

From the perspective of the energy structure and meteorological conditions in south and north China, the north is where China’s heavy industry is concentrated and where winter is cold. Therefore, a robust demand for coal consumption exists, resulting in a low sensitivity to the impacts of cultural diversity and environmental regulations on carbon emissions. However, the south has many service and high-tech industries, and the demand for energy consumption is diversified. Moreover, forming local convective weather conditions and diffusing pollutants is easy because of the mountainous terrain in the south. Thus, a high sensitivity to the impacts of cultural diversity and environmental regulations on carbon emissions was observed.

### 3.4. Mechanism Analysis

As pointed out in the theoretical analysis in the previous section, differences were found in the energy consumption requirements and environmental preferences of micro subjects as reflected by cultural diversity, which may further affect carbon emissions through environmental regulation policies. The empirical results also revealed that cultural diversity and environmental regulations have a combined adverse impact on carbon emissions. In this section, we further investigated the mechanism of cultural diversity influencing carbon emission effects through the mediation of environmental regulations. We used the mediation effect test procedure proposed by Wen et al. [44] to identify the mediation effect of environmental regulations through which cultural diversity impacts carbon emission effects. The test model was as follows:

(5) $$\ln coe_{\mathrm{it}}=\alpha +\beta_{1}\ln dc_{\mathrm{it}}+\beta_{2}\ln\chi_{\mathrm{it}}+\varepsilon_{\mathrm{it}},$$

(6) $$\ln ger_{\mathrm{it}}=\alpha +\gamma_{1}\ln dc_{\mathrm{it}}+\gamma_{2}\ln\chi_{\mathrm{it}}+\varepsilon_{\mathrm{it}},$$

(7) $$\ln coe_{\mathrm{it}}=\alpha +\lambda_{1}\ln ger_{\mathrm{it}}+\lambda_{2}\ln dc_{\mathrm{it}}+\lambda_{3}\ln\chi_{\mathrm{it}}+\varepsilon_{\mathrm{it}}.$$

The definitions of variables in the formulas above are consistent with those in the preceding section, and the test results of the mechanism are presented in Table 6.

According to the empirical results in Table 6, coefficient *β*_1_ in Model (5) was 0.237 and significant at the l% level. According to the Sobel test procedure, *γ*_1_ and $\lambda_{1}$ must be further verified. The coefficients in Model (7) was significant at the 1% level. The Sobel test results were significant at 1% level, indicating the mediation effect of environmental regulations. Cultural diversity has a negative intermediary effect on carbon emission effects through the mediation of environmental regulations.

We discuss the possible practical reasons behind the influence mechanism, which may be due to the cultural diversity embodying energy demand and the environmental preference differences decreasing the intensity of government environmental regulation. Alternatively, the widespread existence of “a few people or organizations make claims, others benefit together” and the traditional Chinese culture’s “benefit protection” philosophy produced a stronger environmental free-riding phenomenon, and there are negative incentives for carbon emission “free-riding” among different cultural groups [42,43]. For other reasons, a greater cultural diversity makes it more unfavorable for the implementation of government environmental regulations. The cultural diversity of micro subjects weakens the effect of the government in controlling carbon emissions through environmental regulation, and the optimal choice of a “rational man” has a significant negative impact, which increases the difficulty of constructing the government–society–public multi-party system of environmental governance.

## 4. Conclusions

This study empirically examined the impacts of cultural diversity on carbon emissions from the perspective of environmental regulations. We found that cultural diversity has a significant impact on carbon emissions. On the one hand, cultural diversity is not conducive to reducing carbon dioxide emissions because differences in citizens’ environmental and energy consumption preferences caused by cultural diversity may weaken the identification of the public with a social emission reduction effect, thereby undermining the realization of carbon emission targets through environmental free-riding behavior. On the other hand, cultural diversity and environmental regulations have a combined negative impact on carbon emissions. Through an analysis of specific mechanisms, the cultural diversity of micro subjects clearly weakens the capability of the government to control carbon emissions through environmental regulations, and it heightens the difficulty of building a government–society–public environmental governance system.

The research conclusions can provide important policy implications for environmental governance.

Firstly, attention should be paid to the important role of culture and other non-institutional factors in the practice of carbon emission reduction. Our study found that non-institutional factors based on cultural diversity have a non-negligible inhibitory effect on carbon emission reduction. In regions with huge cultural diversity, “top-down” equalitarian and all-embracing policies can reduce the effect of carbon emission reduction measures. Thus, the identification and participation of the social public in carbon emission reduction must be strengthened when formulating and implementing emission reduction policies. Moreover, intra-regional differences should be considered to formulate different environmental regulation policies in line with the characteristics of different regions for differentiated governance. Furthermore, the “equalitarian” implementation method must be avoided.

Secondly, focus should be given to the demand feedback of micro subjects in the process of making environmental regulation policies. We attempted to discuss the influence of micro subjects in environmental regulations from the perspective of non-institutional factors. Through a test of the mediation effect of environmental regulations on the impact of cultural diversity on carbon emissions, we found that the cultural diversity of micro subjects generates an adverse effect on carbon emissions through environmental regulations. That is, we fail to fully consider the differences of micro subjects in the formulation of environmental regulation policies. As a result, the effect of environmental regulations on carbon emission reduction is discounted in the implementation of such regulations. Therefore, attention should be paid to perfecting the expression channels of public demand during the formulation and implementation of environmental regulation policies and to the incentives for the environmental protection of micro subjects. A perfect government–society–public environmental governance system, which reflects the government’s role in leadership and planning and the differences between different regions and individuals, must be constructed.

Finally, differentiated governance should be implemented according to the differences in various types of environmental regulations. We confirmed the positive significance of different types of environmental regulation policies in lowering carbon emissions. Although these types have different effects on diminishing carbon dioxide emissions, all policy types have positive roles in reducing carbon dioxide emissions. Micro subjects’ energy consumption behavior must be further strengthened and guided, while the carbon emission market, environmental protection tax, and standards for pollution discharge fees should be further optimized and advanced. Although the influence of administrative regulation is relatively small, its pertinence and timeliness should be improved because of its irreplaceable regulatory role in practice. Such an improvement can further facilitate the realization of carbon emission reduction targets.

## Figures and Tables

**Table 1:** Descriptive statistics of main variables.

Variable	Definition	Units of Measurement	Mean	SD	Min	Max	Med
*coe*_it_	Carbon emission	Tons per person	7.0	4.1	2.3	25.0	5.9
*ger*_it_-*E*	Economic regulation	%	5	5	0.2	51.2	4.1
*ger*_it_-*A*	Administrative regulation	Times/hundred million yuan	0.23	0.35	0.01	2.75	0.12
*ger*_it_-*N*	Energy regulation	Ten thousand tons of standard coal/hundred million yuan	1.31	0.71	0.40	4.15	1.11
*dc*_it_	Cultural diversity		9.0	4.8	1.0	17.5	8.0
*gdp*_it_	Economic development level	Ten thousand yuan per person	46,702	36,528	6339	25,6206	45,342
*dp*_it_	Population density	Person/square kilometer	451	659	8	3847	462
*ins*_it_	Industrial structure	%	47	8	19	59	49
*ecs*_it_	Energy consumption structure	%	62	18	10	93	63

**Table 2:** Regression results of the basic model.

Variable	Extended Name	*ger*_it_-*N*	*ger*_it_-*E*	*ger*_it_-*A*
*Coe*_it-1_	Carbon emission	1.031 ***	0.473 ***	0.872 ***
		(9.59)	(8.41)	(9.67)
*ger*_it_	Environmental regulations	−0.304 ***	−0.102 **	−0.023
		(−3.12)	(−2.01)	(−1.84)
*dc*_it_	Cultural diversity	0.072	−0.083 ***	0.016
		(1.63)	(−3.74)	(0.53)
*gdp*_it_	Economic development level	0.106 **	0.125 ***	0.096
		(2.07)	(2.72)	(1.82)
*dp*_it_	Population density	0.057	−0.121 ***	−0.032
		(1.89)	(−6.84)	(−1.34)
*ins*_it_	Industrial structure	−0.258 **	0.459 ***	0.024
		(−2.10)	(4.04)	(0.27)
*ecs*_it_	Energy consumption structure	−0.003	0.103 ***	−0.012
		(−0.08)	(3.33)	(−0.30)
*cons*	Constant	−0.826	0.886 **	−0.160
		(−1.66)	(2.39)	(−0.37)
Individual effect		Yes	Yes	Yes
Time effect		Yes	Yes	Yes
Ar (1)		−3.45	−4.34	−4.78
Ar (1)-p		0.001	0.000	0.000
Ar (2)		−0.72	−0.28	−0.06
Ar (2)-p		0.471	0.779	0.955
Sargan-p		0.773	0.391	0.200
N		245	245	245

Note: ** and *** indicate significance at 5% and 1% significance levels, respectively. The *t* statistic is in parentheses. AR indicate the result of autocorrelation test.

**Table 3:** Panel Granger causality test results.

Hypothesis	F	Policy Decision
Cultural diversity is not the Granger cause of carbon emissions	2.8595	Reject the original hypothesis
	(0.0251)	
Carbon emissions are not the Granger cause of cultural diversity	0.0432	Accept the original hypothesis
	(0.9964)	

Note: F indicates the F statistic. The *p* statistic is in parentheses.

**Table 4:** Empirical results of interaction terms (cultural diversity and environmental regulations).

Variable	Extended Name	*ger*_it_-*N*	*ger*_it_-*E*	*ger*_it_-*A*
*coe*_it−1_	Carbon emission	0.968 ***	−0.402 ***	−0.987 ***
		(10.24)	(−0.07)	(6.89)
*ger*_it_	Environmental regulations	−2.86 **	−0.062 ***	0.294 **
		(−3.03)	(−2.66)	(2.06)
*ger*_it_ * × dc*_it_	Environmental regulations × Cultural diversity	1.309 **	0.322 **	0.149
		(3.03)	(2.41)	(1.97)
*dc*_it_	Cultural diversity	0.004	2.917 **	2.08 **
		(0.13)	(2.50)	(2.39)
*gdp*_it_	Economic development level	−0.327 **	0.780 **	0.680 ***
		(−2.62)	(2.55)	(9.51)
*dp*_it_	Population density	−0.065 **	−0.225 **	−0.412 ***
		(−1.66)	(−2.23)	(−12.25)
*ins*_it_	Industrial structure	0.573 **	−0.379 **	1.208 ***
		(2.48)	(−2.09)	(8.63)
*ecs*_it_	Energy consumption structure	−0.123	0.342 **	−0.403 ***
		(−1.92)	(2.31)	(7.85)
*cons*	Constant	1.625 **	6.272 ***	8.915 ***
		(2.74)	(2.69)	(4.64)
Individual effect		Yes	Yes	Yes
Time effect		Yes	Yes	Yes
Ar (1)		−2.33	0.78	4.74
Ar (1)-p		0.008	0.436	0.000
Ar (2)		−0.020	0.47	−0.84
Ar (2)-p		−0.71	0.638	0.400
Sargan-p		0.479	0.808	0.212
N		245	245	245

Note: ** and *** indicate significance at 5% and 1% significance levels, respectively. The *t* statistic is in parentheses. AR indicate the result of autocorrelation test.

**Table 5:** Empirical results of north and south China.

Variable	Extended Name	North			South		
		*ger*_it_-*N*	*ger*_it_-*E*	*ger*_it_-*A*	*ger*_it_-*N*	*ger*_it_-*E*	*ger*_it_-*A*
*coe*_it−1_	Carbon emission	0.944 ***	0.990 ***	0.924 ***	1.132 ***	0.394 **	0.337 ***
		(8.34)	(10.78)	(10.83)	(5.51)	(1.99)	(5.25)
*ger*_it_	Environmental regulations	−0.423	0.224	−0.041	−0.650	0.233 ***	−0.005 **
		(−1.31)	(1.89)	(−1.12)	(−1.54)	(3.16)	(−2.29)
*ger*_it_ × *dc*_it_	Environmental regulations × Cultural diversity	0.277	−0.122	0.023	0.286	−0.036	−0.046 **
		(1.46)	(−1.66)	(1.10)	(1.79)	(−1.97)	(−2.13)
*dc*_it_	Cultural diversity	−0.134	−0.891	0.271	0.075 **	0.367 **	0.604 **
		(−1.56)	(−1.66)	(1.15)	(1.99)	(2.18)	(2.44)
*gdp*_it_	Economic development level	−0.017	0.061	0.051	−0.098	0.394 ***	0.323 ***
		(−0.13)	(0.61)	(0.59)	(−0.73)	(3.15)	(9.97)
*dp*_it_	Population density	−0.015	−0.023	−0.034	−0.042	−0.030 ***	−0.030 ***
		(−1.51)	(−1.68)	(−1.74)	(−1.13)	(−4.07)	(−6.32)
*ins*_it_	Industrial structure	0.186	−0.128	0.084	0.054	−0.052 **	0.154 ***
		(1.55)	(−1.34)	(1.23)	(0.96)	(−1.77)	(4.95)
*ecs*_it_	Energy consumption structure	0.198	−0.126	0.032	−0.035	0.054 ***	0.061 ***
		(1.78)	(−1.06)	(1.37)	(−0.84)	(3.60)	(5.04)
*cons*	Constant	−0.255	2.127	−0.282	0.229	2.564 ***	1.581 ***
		(−1.23)	(1.79)	(−0.65)	(1.36)	(3.19)	(5.16)
Individual effect	-	Yes	Yes	Yes	Yes	Yes	Yes
Time effect	-	Yes	Yes	Yes	Yes	Yes	Yes
Ar (1)	-	−3.41	−3.74	−3.47	−3.28	−1.68	−2.65
Ar (1)-p	-	0.001	0.000	0.001	0.001	0.092	0.008
Ar (2)	-	−0.59	−0.81	−0.69	−0.97	−0.83	−0.74
Ar (2)-p	-	0.556	0.416	0.491	0.333	0.405	0.462
Sargan-p	-	0.253	0.633	0.244	0.798	0.762	0.331
N	-	126	126	126	144	144	144

Note: ** and *** indicate significance at 5% and 1% significance levels, respectively. The *t* statistic is in parentheses. AR indicate the result of autocorrelation test. The southern region includes 16 provinces, namely, Shanghai, Jiangsu, Zhejiang, Anhui, Fujian, Jiangxi, Henan, Hubei, Hunan, Guangdong, Guangxi, Hainan, Chongqing, Sichuan, Yunnan, and Qinghai; the northern region includes 14 provinces, namely, Beijing, Tianjin, Hebei, Shanxi, Inner Mongolia, Liaoning, Jilin, Heilongjiang, Shandong, Henan, Shaanxi, Gansu, Ningxia, and Xinjiang.

**Table 6:** Test of the impact mechanism: mediation effect of environmental regulations.

Variable	Extended Name	Model (5)	Model (6)	Model (7)
*dc*_it_	Cultural diversity	0.237 ***	−0.075	0.226 ***
		(5.65)	(−1.78)	(5.39)
*coe*_it−1_	Carbon emission	−0.015	0.002	−0.015
		(0.28)	(0.03)	(−029)
*ger*_it_	Environmental regulations			0.156 ***
				(2.59)
*gdp*_it_	Economic development level	0.112 ***	−0.078	0.124 ***
		(2.65)	(−1.84)	(2.95)
*dp*_it_	Population density	−0.176 ***	−0.214 ***	−0.143 ***
		(−6.77)	(−8.17)	(−4.96)
*ins*_it_	Industrial structure	0.645 ***	0.483 ***	0.569 ***
		(4.94)	(3.69)	(4.30)
*ecs*_it_	Energy consumption structure	0.192 ***	−0.077	0.204 ***
		(3.04)	(−1.23)	(3.26)
*cons*	Constant	2.928 ***	2.280 ***	2.571 ***
		(7.71)	(5.99)	(6.43)
Sobel test		Z = −2.441, P = 0.014		

Note: *** indicate significance at 1% significance levels, respectively. The *t* statistic is in parentheses.
